# Global mental health: an interview with Vikram Patel

**DOI:** 10.1186/1741-7015-12-44

**Published:** 2014-03-14

**Authors:** Vikram Patel

**Affiliations:** 1 Centre for Global Mental Health, London School of Hygiene, Keppel Street, London WC1E 7HT, UK; 2 Sangath, Bhatkar Waddo, Succour, Porvorim, Bardez, Goa 403501, India; 3 Centre for Mental Health, Public Health Foundation of India, ISID Campus, 4 Institutional Area, Vasant Kunj, New Delhi 110070, India

## Abstract

In this podcast, we talk to Professor Vikram Patel about the impact of global mental health in the field of medicine, and discuss the initiatives and platforms being developed to promote capacity building, research, policy and advocacy within the established Centre for Global Mental Health. The anticipated challenges, controversies, and future directions for this discipline of global health are highlighted as well.

The podcast for this interview is available at: http://www.biomedcentral.com/sites/2999/download/Patel.mp3.

## Introduction

**Figure 1:**
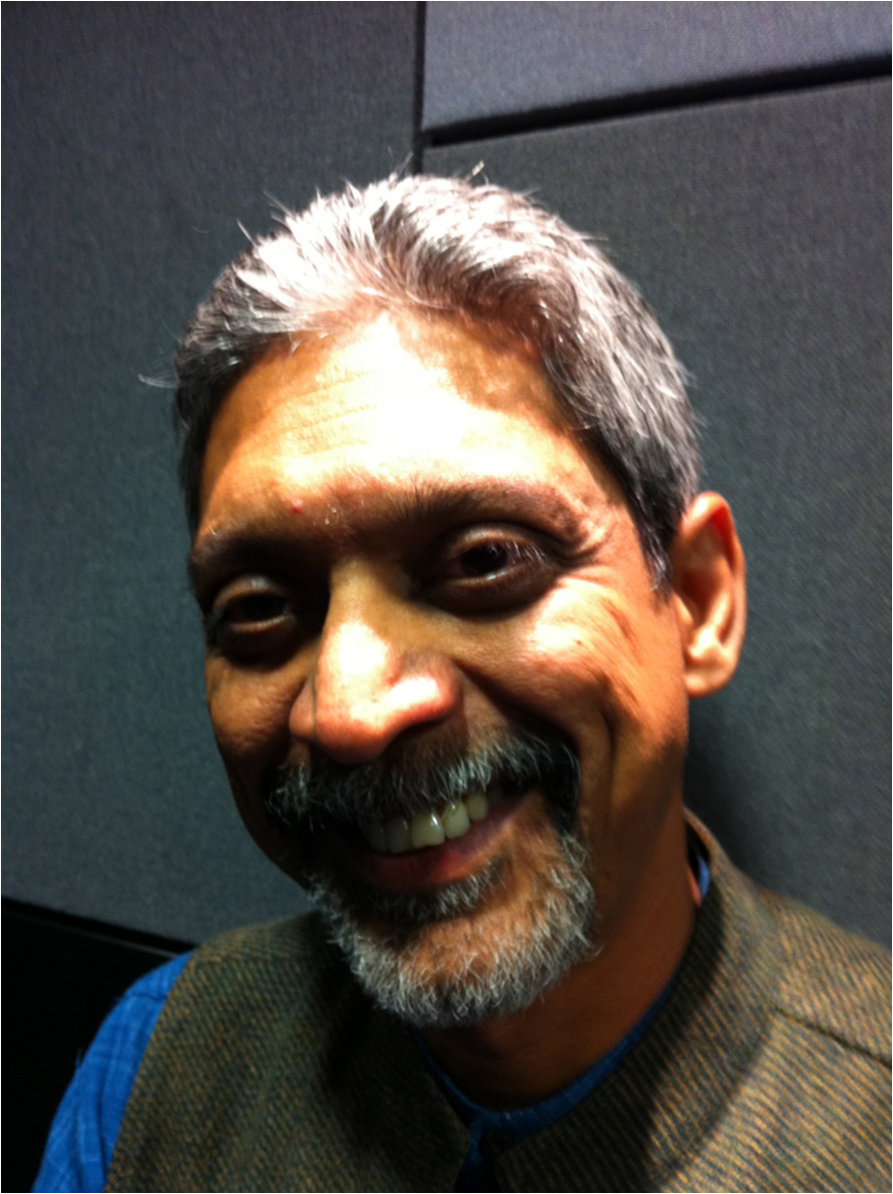
Vikram Patel.

Professor Vikram Patel is a psychiatrist who works for the London School of Hygiene and Tropical Medicine, where he is the joint Director of the Centre for Global Mental Health. He has held a Wellcome Trust research fellowship now for nearly 15 years, with about 9 of these years being as a senior research fellow in clinical science. For most of the year, he is based in India, where he works with a community-based research organization called Sangath, which he co-founded about 17 years ago. He also works with the Public Health Foundation of India, where he recently took on the position as the Honorary Director of the Centre for Mental Health in New Delhi (Figure 1).

The podcast for this interview is available at: http://www.biomedcentral.com/sites/2999/download/Patel.mp3.

## Edited transcript

### What is global mental health and how will it impact the field of medicine?

Global mental health is a discipline of global health and, as with the mission of global health, its primary goal is to improve the health of people worldwide, with a strong focus on equity and access. There is no health without mental health. I think we know, through a large body of evidence, that mental health and physical health interact with each other in very diverse and intimate ways. Therefore, any attempt that we make to improve the mental health of individuals and populations will inevitably have a positive impact on the physical health of those individuals and populations. Therefore, investing in global mental health is, ultimately, an investment in global health.

### How was the Centre for Global Mental Health established?

The Centre for Global Mental Health is a partnership between the London School of Hygiene and Tropical Medicine, which is Europe’s leading school of public health, and the Institute of Psychiatry at Kings Health Partners, which is Britain’s leading school of psychiatry and neurosciences. It’s a perfect marriage between academics who have strengths in the various disciplines of global health, and academics who have strengths in the various disciplines of clinical sciences related to mental health. The centre was founded in 2008 to bring these two complementary sets of disciplines on to the same platform to further the science of global mental health. The goal was to promote research, capacity building, and advocacy for policy to improve access to care for people living with mental disorders around the world, with a particular focus on those countries where the treatment gaps were the largest; that is, the low- and middle-income countries of the world.

### Can you describe the initiatives and platforms that are involved with this collaboration?

Let me give you examples from each of the three broad themes of work that the Centre for Global Mental health pursues. Firstly, with capacity building, we are delighted that, after many years of plotting and planning, we were able to launch our full-time Masters in Global Mental Health last year. This year, we have had more than 20 applicants successfully admitted and beginning the MSc program. The MSc is, to my knowledge, the only face-to-face residential MSc in this discipline in the world today.

In the area of research, we are currently involved with dozens of research projects in more than 20 countries within sub-Saharan Africa, Latin America and Asia. Some of this research focuses on randomized controlled trials of innovative new interventions to improve access to evidence-based treatments, but we are also engaged in some exciting health systems work that examines how these evidence-based packages of care can be integrated into routine healthcare systems. Our portfolio also includes social science and epidemiological research on mental health problems.

Finally, in the realm of policy and advocacy, we have recently embarked on a number of exciting developments. The first is that we led the Mental Health Forum for the World Innovation Summit for Health, which was held in Qatar in December 2013. Along with Shekhar Saxena of the World Health Organization, I co-chaired this forum, which produced a report specifically directed at ministers of health and other policymakers, to recommend policy actions based on the research evidence in global mental health [1]. Another exciting development is the Mental Health Innovations Network [2], supported by Grand Challenges Canada, whose goal is to synthesize the rapidly growing evidence base in the field into products that can be useful to a variety of different audiences, from researchers and practitioners to civil society and policymakers.

### This is very exciting in terms of all the initiatives and platforms. What do you think are the current anticipated challenges?

There are a number of different challenges. The key is limited resources. There has actually been a fantastic increase in the amount of resources available for research, and of course the Centre for Global Mental Health has been a great beneficiary of that largesse. However, there has not been a similar increase in resources for mental health for ministries of health, particularly in the poorest countries of the world, which rely, to a large extent, on development assistance for their health programs. Thus there has not been the needed increase in resources to scale up mental health services in these countries. So the first important challenge is to mobilize development agencies to finance mental health services in the poorest countries of the world.

The second important challenge is a continuing concern among some communities about the validity of some of the mental health problems that the field is grappling with, in particular the common mental health problems like depression and anxiety. The concern is really whether these conditions are biomedical categories that have universal validity in all cultures, and whether the biomedical approaches that are being utilized in medicine and psychiatry in particular, are relevant and appropriate to all cultures of the world.

### Can you indicate also the controversies such as the debate against global mental health?

In fact, my second point is at the heart of the critique that certain mental illness categories, such as depression in particular, do not travel well across cultures. The critique is that the use of such labels represents a medicalization of a social condition where the solutions lie not within a medical approach but more likely within the social or political sphere. And related to this is the concern of exporting psychiatric paradigms of treatment and care which have been at the heart of the mental healthcare systems of the developed world to developing countries where there is very little formal psychiatric care.

### Do you also think that global mental health will be influenced by DSM-5 with its recent launch, and ICD-11, which will be launched in the future?

The honest truth is that global mental health is a completely different animal from its predecessor, which comprised cultural psychiatry and international psychiatry. First of all, global mental health is not simply psychiatry. Like global health, it is an interdisciplinary endeavor, and is firmly grounded in the South (that is, in the developing world). Most of the leading practitioners of global mental health live and work in developing countries, not in the developed world. Global mental health is completely contextualized to the cultural and social circumstances of the country in which this work is being carried out, and is action-oriented, seeking to improve the lives of people affected by mental health problems.

An important implication of this reality, in relation to your question, is the replacement of rigid diagnostic systems, which are much more suited to psychiatry and the specialized mental healthcare systems you might encounter in developed countries, with broader, more public health-oriented and contextually appropriate labels and diagnostic systems that communicate better to local policymakers, primary care workers and most importantly, to local communities. Global mental health barely uses DSM-4 or ICD-10 in any concrete way, and I think it is unlikely that DSM-5 or ICD-11 will have much traction either.

### What do you think are the future directions for global mental health?

The future directions of global mental health lie in three big areas. The first is to mobilize resources by advocating to policymakers, especially in middle-income countries which have more resources, to finance scaling up of mental health care. This is particularly important in the context of universal health care to advocate for mental health to be given at least parity with physical health in resource allocation and service provision in middle income countries. For low income countries that continue to be dependent on development assistance, we need to be similarly advocating with donors to increase their resources specifically for mental health.

The second is to build capacity. It has to be admitted that there is a great shortage of every kind of mental healthcare provider in the developing world, from specialists like psychiatrists and psychologists all the way through to community-based workers who can provide frontline mental healthcare. There is a great need for investing both in programs that can build capacity that is scalable, and in curricula and other kinds of tools that can be utilized in these sorts of settings for these diverse professional groups.

The third, of course, is research. We clearly need to continue to build evidence which focuses on addressing questions about how we can integrate evidence-based packages for care within routine healthcare systems, so that we can inform governments on how they can make their mental health programs more effective and efficient.

### What are you most excited about in relation to the recent developments in global mental health?

What I am really most excited about is that mental health has come out of the closet. I remember 15 years ago when I began working in this field, it was usually embarrassing in India to say that you were a psychiatrist because, if they did not walk away from you, they would look at you perplexed and ask, “Is this really relevant in our country?” I think there has been a dramatic change in the attitudes towards mental health in every sector of society in India, which is the country I know best, whether it is in the community, in the media in terms of the amount and the quality of the reporting on mental health issues, and of course at the level of policymakers. Today, it is so straightforward for me to sit with a Secretary of Health and talk about mental health issues; they are much more receptive, and indeed, more importantly, are much more willing to back their interest with resources.

### Where can I find out more?

See references [3-12].

## Competing interests

VP is a full time staff of the LSHTM, and receives salary support from the Wellcome \trust. He is also a recipient of grants from the Wellcome Trust, National Institute of Mental Health (NIMH), Autism Speaks, the Sir Dorabji Tata Trust, the Macarthur Foundation and the Department for International Development (DFID). He is the lead editor of the recent Oxford University Press textbook on global mental health.
